# Data-driven design of LNA-blockers for efficient contaminant removal in Ribo-Seq libraries

**DOI:** 10.1038/s41598-026-43117-3

**Published:** 2026-03-09

**Authors:** Dario A. Ricciardi, Franziska E. Peter, Maik Böhmer

**Affiliations:** Institute of Molecular Biosciences, Plant Physiology, University of Frankfurt, Max-von-Laue Str. 9, 60438 Frankfurt am Main, Germany

**Keywords:** Biological techniques, Biotechnology, Computational biology and bioinformatics

## Abstract

Ribo-Seq libraries often contain highly abundant non-coding RNA contaminants, which are challenging to remove due to their high sequence variability and diverse fragmentation patterns. We present an organism-independent computational pipeline that identifies experiment-specific target sequences and enables their efficient depletion using custom-tailored LNA probes in a single pipetting step. We demonstrate that LNA-based depletion is most effective during library amplification and has no effect on gene-level quantification. Contaminant depletion in Arabidopsis libraries nearly doubled the yield of coding reads, significantly improving cost-effectiveness.

**Supplementary Information:**

The online version contains supplementary material available at 10.1038/s41598-026-43117-3.

## Introduction

Ribosome profiling (Ribo-Seq) is a relatively new method that enables the quantification of regulatory events at the translational level, similar to RNA-Seq at the transcriptional level^1^. For a Ribo-Seq analysis, ribosomes are extracted from tissue in their native state, still bound to actively translated mRNA transcripts. Exposed mRNA not protected by the ribosome is degraded by nuclease digestion, while the ribosome-protected fragments (RPFs), or ribosomal footprints, are purified and sequenced. RPFs are then mapped back to the reference genome to reveal the exact distribution of translating ribosomes, producing a snapshot of the translatome. Especially when combined with transcriptomic data, this information can be used to infer translation efficiency, slow/pause sites, and alternative reading frames, making Ribo-Seq an essential technique for understanding translational regulation^2^.

The steps to process RPFs often result in Ribo-Seq datasets containing a significant amount of contaminant fragments of non-coding origin that co-purify in the same size range as RPFs (Supplementary Figure S1). Established rRNA mitigation strategies that rely on probes with pre-defined target sequences underperform when applied to Ribo-Seq samples due to the fragmentation of their targets^3^. Additionally, the contaminant profile is defined by the organism, growth and experiment conditions, and the nuclease used for footprint generation, resulting in numerous possible contaminant combinations^4^.

The specificity and therefore the efficiency of the depletion have been improved significantly by using custom-tailored probes, coupled with affinity purification^5–9^, nuclease digestion^10–12^, or Cas 9 cleavage^13,14^. However, these methods require considerable hands-on time, incubation steps, and, especially, purification steps, which can lead to sample loss and bias.

Oligonucleotides containing locked nucleic acids (LNA), which are chemically modified nucleotides with a fixed conformation that enhances binding affinity, can effectively bind contaminant fragments during library preparation and sterically hinder reverse transcription or amplification, eliminating the need for additional cleanup steps^15^.

We present a workflow to efficiently remove contaminants from Ribo-Seq libraries in a single pipetting step using LNA oligonucleotides. The included R script identifies and visualizes experiment-specific contaminants from an initial sequencing run and provides optimized LNA target sequences. Additionally, we supply contaminant profiles for common growth conditions of *Arabidopsis thaliana* plants.

A preliminary sequencing experiment with a smaller sample size and reduced sequencing depth is conducted under the planned experimental conditions to identify the various contaminant RNA species generated by ribonuclease activity during footprint formation. This enables the characterization of contaminant fragment profiles specific to the experimental setup. We developed an R script that utilizes pre-aligned reads from the small-scale sequencing run to define groups of experiment-specific contaminants based on sequence similarity. The shortest common sequence in each group is reported as the optimal LNA target sequence, along with a heatmap of contaminant fragment distribution across all samples and a figure of merit to help the user gauge expected depletion performance relative to the number of LNA targets. Using our optimized sequence aggregation process, small datasets (10–20 million total reads) can be analyzed within minutes on modern hardware, independent of the host organism, provided the necessary annotations are available in GTF or GFF format.

**Fig. 1:**
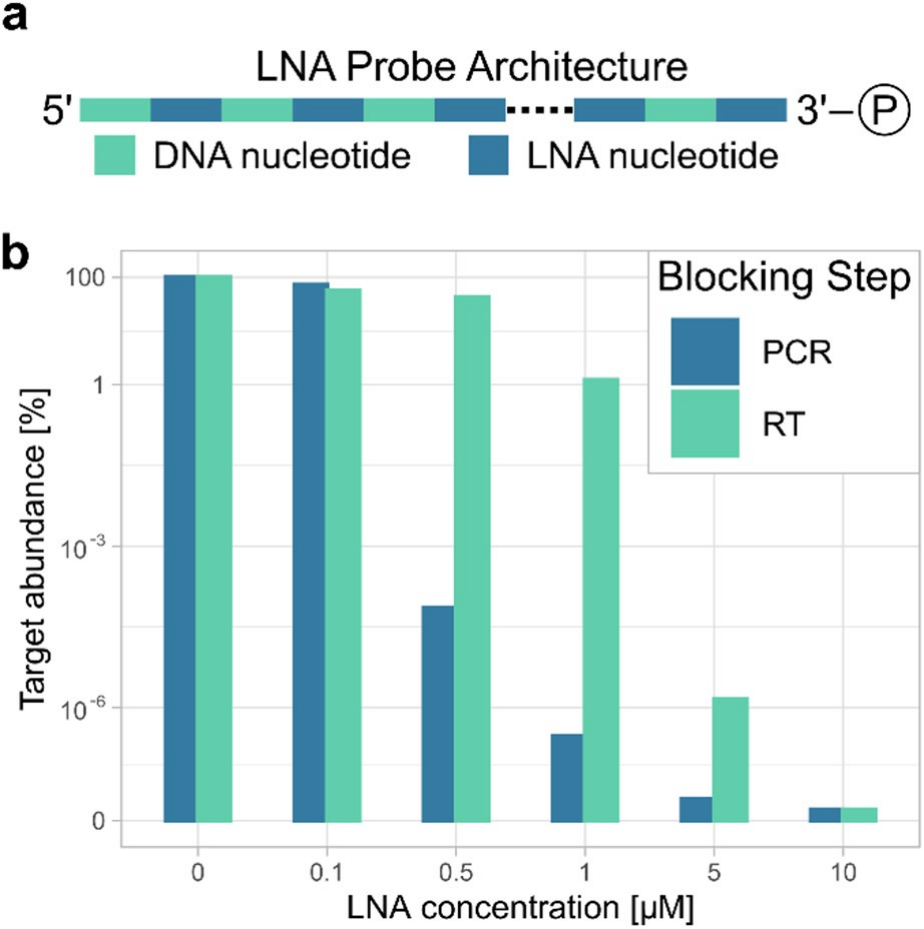
LNA design and application considerations. (**a**) Optimal architecture of LNA probes. Alternating DNA and LNA nucleotides, beginning with a DNA nucleotide. The dotted line represents variable length. Phosphate residue at the 3’-end. (**b**) Blocking performance of a single LNA against its target (fragment of 5.8 S rRNA) at various concentrations. Comparison between blocking at the reverse transcription step and during PCR amplification. Target abundance percentages are based on qPCR cycle difference in relation to the non-depleted sample (0 µM).

For optimal performance, LNA oligonucleotides should consist of alternating DNA and LNA nucleotides, starting with a DNA nucleotide^15^. For Arabidopsis, we recommend a minimum length of 14 nucleotides, primarily due to sequence specificity concerns. Other organisms may require longer oligos depending on genome size. Standard primer design considerations apply here. Additionally, phosphorylation of the 3’-end prevents the LNA oligonucleotides from acting as primers themselves and producing a truncated amplicon that could interfere with downstream analysis (Fig. 1a).

LNAs can be used to block either the reverse transcription or the PCR amplification of the library. To identify the best step, we evaluated the depletion efficiency using primers for the top contaminant and added increasing concentrations of the appropriate LNA probe at each step. (Fig. 1b). RT-qPCR was employed to measure the abundance of the target sequence in the Ribo-Seq library. Using a final concentration of 0.5 µM of the LNA oligonucleotide in the PCR mix reduced the level of the targeted contaminant by more than 1000-fold. Blocking at the reverse transcription step required at least twice the concentration to achieve similar results. Adding the LNA probes during amplification offers the benefit of strand independence. Blocking either the forward or reverse product effectively decreases amplification efficiency, making LNA probe design easier by removing the need to match the template sequence during reverse transcription.

**Fig. 2:**
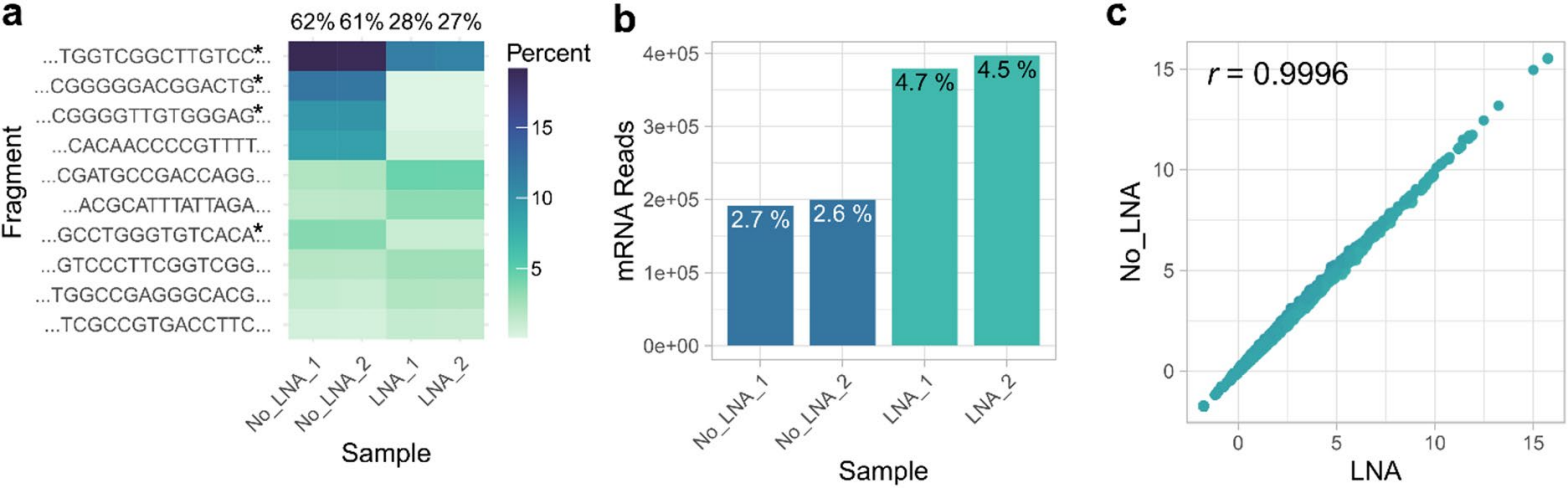
Depletion performance of five LNAs targeting the most abundant contaminants. (**a**) Heatmap visualization of undepleted samples and samples that were supplemented with an LNA mixture during library amplification. The LNA mix contained five probes at 1 µM each, targeting the most abundant contaminants identified across all tested growth conditions. The shortest common sequence of each contaminant group is indicated on the y-axis; LNA target sequences are marked with an asterisk. Tile color represents the percentage of that group in relation to the total reads in the sample. Cumulative percentages for all displayed contaminants are summarized at the top of each column. (**b**) Comparison between the amount of mRNA reads in undepleted and LNA-depleted samples. Percentages in relation to total read count per sample are indicated at the top of each bar. (**c**) Correlation of averaged gene-level read counts between undepleted and depleted samples. Counts were regularized log-transformed using the “rlog” function from the DESeq2 R package. Libraries were processed with the ORFik toolkit^16^, filtering for reads over 20 nt and removing reads that mapped to non-coding RNAs. Pearson correlation coefficient is denoted in the top left corner.

After optimizing the concentration and application of the LNA blockers, we designed a set of five LNAs targeting the top five contaminant fragments across various common growth conditions of *Arabidopsis thaliana* (Supplementary Figure S2), providing a ready-to-use alternative to custom probe sets (tables for each growth condition are available in the GitHub repository). Pooled RPF extracts from hydroponically grown, untreated wild-type Arabidopsis seedlings were split and converted into four identical sequencing libraries. LNA blockers were added to two libraries during the amplification stage at a concentration of 1 µM each. The resulting reads were aligned and analyzed with our script. Targeted depletion of the top five contaminants reduced the total identified contaminants by over 30% (Fig. 2a). Sequences targeted by the blockers were eliminated considerably. At the same time, the yield of RPFs of coding origin nearly doubled (Fig. 2b). Only four of the five LNA sequences appear in the heatmap. LNA3 targeted a smaller set of contaminants and therefore does not appear among the top 10 most abundant groups in the heatmap. To verify that the depletion did not adversely affect read counts from mRNA transcripts of interest, we correlated the rlog-transformed average counts from undepleted libraries with those from LNA-depleted libraries, considering only mRNA reads over 20 nt that do not map to non-coding features (Fig. 2c). We observed a near-perfect correlation between the two datasets (Pearson correlation coefficient = 0.9996), supporting the notion that LNA depletion does not affect gene-level quantification.

Since Ribo-Seq is an inherently complex technique that poses significant challenges for researchers aiming to decipher translational regulation, we have developed a user-friendly workflow that eliminates the need for extensive bioinformatics expertise and provides comprehensive guidance on the design and application of effective LNA probes. Additionally, consolidating contaminant depletion into a single step enhances the method’s accessibility, particularly for researchers who wish to initiate Ribo-Seq experiments.

The targeted LNA-based depletion can also be integrated into established workflows, offering greater flexibility and cost-effectiveness compared to bead-based approaches. Aside from the provided contamination identification script, other established Ribo-Seq analysis pipelines can be used to infer target sequences and design LNA probes according to our guidelines. Though LNA probes are more expensive than pure DNA oligonucleotides, they often amortize after the first use, considering the increase in effective sequencing depth of RPFs. Depletion with a small set of LNAs nearly doubled the yield of RPFs in our tests, which would otherwise require twice the sequencing depth.

The flexible nature of our approach lends itself to combination with other strategies, such as Ribo-FilterOut^17^ or the rRNA-avoiding nuclease P1^18^, to benefit from possible synergistic effects. Future endeavors could expand our initial set of contaminants by establishing well-defined, cross-validated target sets for specific organism-nuclease combinations across various tissues and conditions, thereby serving as an information resource for the entire Ribo-Seq community.

## Materials and methods

### Biological material

All experiments were conducted using wild-type *Arabidopsis thaliana* (Col-0, NASC ID: N1093) cultivated at the University of Frankfurt. To assess how growth conditions affect the contaminant profile, Arabidopsis plants were grown in soil for either 8 days or 3 weeks, under long-day (LD, 16 h of light) and short-day (SD, 8 h of light) conditions in a climate chamber at 19–22 °C. Under the same environmental conditions, seedlings were grown hydroponically for 8 days in ½-strength Murashige-Skoog (MS) medium containing 2.56 mM MES buffer, either with or without 1% (w/v) sucrose. Etiolated seedlings were grown hydroponically in ½-strength MS medium with sucrose in total darkness after a brief period of illumination to synchronize germination. The seedlings used for testing the effectiveness of LNA depletion were grown hydroponically for 8 days in ½-strength MS medium without sucrose under short-day conditions.

### Purification of ribosomal footprints

Purification of ribosomal footprints was adapted from Hsu et al.^19^ with modifications. In short, plant material (leaf discs for 3-week-old plants, whole seedlings for 8-day-old plants) was harvested into liquid nitrogen, homogenized, and polysomes were extracted by adding 200 µl of ice-cold polysome extraction buffer (100 mM TRIS pH 8.0, 40 mM KCl, 20 mM MgCl_2_, 1% (w/v) sodium deoxycholate or 1% (v/v) Triton X-100, 2% (v/v) polyoxyethylene (10) tridecyl ether, 1 mM DTT, and 355 µM (100 µg/ml) cycloheximide). The mixture was incubated on ice for 5–10 min, then centrifuged at 20,000 g for 7 min at 4 °C. The supernatant was transferred to a new tube and pooled if necessary. The total RNA concentration was determined using a Qubit 4 fluorometer with the RNA Broad Range Assay (Thermo Fisher). Footprints were generated by the addition of 0.3 U RNase If (New England Biolabs) per 1 µg of total RNA in a sample volume of 110 µl and incubation for 30 min at 21 °C with agitation at 300 RPM. Digested extracts were immediately loaded onto pre-equilibrated size-exclusion columns (MicroSpin S-400 h, Cytiva) and spun for 90 s at 750 g to separate monosomes from free RNA fragments. The flow-through containing the monosomes was mixed with TRIzol (Invitrogen) to purify RNA. The resulting RNA pellet was resuspended in PNK reaction buffer (New England Biolabs) containing 10 mM ATP for end repair of the RNA fragments at 37 °C for 30 min. End-repaired RNA fragments were purified by ammonium-acetate/isopropanol precipitation and resuspended in 2x urea loading buffer. The purified RNA was separated on a denaturing 15% polyacrylamide gel containing 7 M urea (TAE buffer) at 100 V for 15 min, then at 150 V for 60 min. Bands in the size range between 18 and 32 nt were excised from the gel and eluted according to Reid, Shenolikar, and Nicchitta^20^. However, instead of the freeze-boil cycles, the homogenized gel pieces were sonicated for 1 h in an ultrasonic bath. The eluted RNA was precipitated by adding one volume of isopropanol and 1.3 µL GlycoBlue coprecipitant (Invitrogen), and incubating for at least 1 h or overnight. The precipitated RNA was washed and resuspended in 7 µl of RNase-free water.

### Library preparation and depletion of contaminants

Purified ribosomal footprints (including contaminants) were quantified with the Qubit 4 fluorometer, using the RNA High Sensitivity assay (Thermo Fisher). Between 5–20 ng of RNA was used as input for library preparation with the TrueQuant SmallRNA Seq kit (GenXPro). According to the manufacturer, when using already purified footprints at these concentrations, dilution of adapters and primers was not necessary. Libraries were prepared according to the manual with overnight incubation of the 5’-adapter, as recommended for plant RNA.

Depletion of contaminant sequences with custom LNA probes was performed during the library amplification step by adding a 10-fold LNA mix to the water volume in the PCR reaction. The LNA mix included five LNA probes (Table 1), each at a concentration of 10 µM, resulting in a final concentration of 1 µM in the master mix. Libraries were amplified for 7 cycles based on preliminary test amplifications. Final library concentrations were measured with the Qubit 4 fluorometer and the 1x DNA High Sensitivity assay (Thermo Fisher).

Sequencing was performed at GenXPro (Frankfurt, Germany), targeting 4–7 million 75 bp single-end reads. Due to the high abundance of contaminating fragments, low sequencing depths are sufficient for preliminary testing. The trimmed reads are available at the European Nucleotide Archive (ENA) under accession number PRJEB94291.

### Data analysis and identification of contaminant sequences

Sequenced reads were quality-controlled with FastQC version 0.12.1^21^ and filtered with seqkit^22^, retaining only reads between 20 and 30 nt to account for variations during gel excision. The filtered reads were aligned to the *Arabidopsis thaliana* reference genome (TAIR 10 release) with the STAR aligner^23^ using the Araport 11 Oct. 2024 release^24^. For accurate quantification of multimapping contaminants, we ran STAR with –outSAMmultNmax 1 and –outFilterMultimapNmax 20 (sufficient for Arabidopsis, may need to be increased for other organisms to ensure inclusion of all multimapping reads). Alignments in BAM format were used as input for our contamination identification script in R (version 4.0 or later). First, aligned reads are mapped to features in the supplied annotation file, and identical reads are collapsed into single reads with counts using the packages Rsamtools^25^, GenomicFeatures^26^, and txdbmaker^27^. The reads are then sorted by their abundance, and the first 10,000 are selected as matching patterns. These reads are sorted by length, and the shortest is matched throughout the entirety of all remaining reads via a “grep” command. All matching reads are grouped, and their summed counts, as well as the shortest common sequence in the set, are extracted as a result. All matched reads are eliminated from the search set before the next shortest read is queued for matching. This increases computation speed. The final data frame, containing the shortest sequence patterns and associated counts, is visualized using the ggplot2 package^28^. We generated a heatmap illustrating the distribution of similar contaminants across all samples, along with a figure of merit showing the aggregate percentage of contaminants targeted by increasing LNA set size. The contaminant identification script is available at https://github.com/ZeroG-lab/LNA-Depletion.

Features for non-multimapping reads were counted using the --quantMode GeneCounts flag in STAR. Exploratory data analysis on the counts was performed in R using the DESeq2 package^29^. Quality control, mRNA species mapping, and generation of non-contaminant read counts were performed with the ORFik toolkit^16^.

### LNA performance testing

We determined the optimal application method of the LNAs in terms of concentration and blocking step by assessing the abundance of targeted transcripts using RT-qPCR. Total RNA was extracted from leaves of 3-week-old *Arabidopsis thaliana* Col-0 wild-type plants using TRIzol reagent (Invitrogen). Purified RNA was reverse transcribed to cDNA with the iScript cDNA Synthesis Kit (Bio-Rad). The template cDNA was amplified using the PowerUP SYBR Green Master Mix in a StepOnePlus Real-Time PCR system (Applied Biosystems). The highly abundant 5.8 S ribosomal RNA was chosen as a target (fwd: CGATGAAGAACGTAGCGAAA; rev: TTGTGACACCCAGGCAG). An LNA probe (CCAGGCAGACGTGCCC) targeted to the 5.8 S rRNA was added in increasing concentrations (0.1 µM, 0.5 µM, 1 µM, 5 µM, 10 µM) at either the reverse transcription step or during the qPCR amplification. For the reverse transcription depletion, 200 ng of template RNA were used in conjunction with the LNAs. For the amplification depletions, 1 µg of template RNA was first reverse-transcribed without LNAs, and then 20 ng of the resulting cDNA was amplified with the LNAs. This 10-fold difference in template concentration between the reverse transcription and amplification steps is practical, as it mimics real-world workflows in which the PCR master mix further dilutes the reverse-transcribed cDNA. Target abundance was calculated from the cycle threshold differences between the tested LNA concentrations.

**Table 1:** Locked nucleic acid (LNA) oligonucleotides used for the depletion of the most abundant contaminants across all Ribo-Seq libraries from *Arabidopsis thaliana* under various growth conditions. LNA nucleotides are denoted in bold letters.

LNA (length [nt])	Sequence
LNA_Top_1 (14)	G**C**C**T**G**G**G**T**G**T**C**A**C**A**
LNA_Top_2 (14)	C**G**G**G**G**G**A**C**G**G**A**C**T**G**
LNA_Top_3 (20)	C**T**G**T**T**T**T**T**G**G**T**C**C**C**A**A**G**G**C**T**
LNA_Top_4 (14)	T**G**G**T**C**G**G**C**T**T**G**T**C**C**
LNA_Top_5 (14)	C**G**G**G**G**T**T**G**T**G**G**G**A**G**

## Supplementary Information

Below is the link to the electronic supplementary material.

Supplementary Material 1

## Data Availability

In accordance with the FAIR Guiding Principles, all data and code underlying this study have been made publicly available. The R script used for data processing and analysis, along with detailed contaminant tables for each condition, is available on GitHub at https://github.com/ZeroG-lab/LNA-Depletion. The next-generation sequencing data generated in this study have been submitted to the EMBL (ENA) database under accession number PRJEB94291.
