# Supplementary material for: Data-driven design of LNA-blockers for efficient contaminant removal in Ribo-Seq libraries
Source: Sci Rep. 2026 Mar 9;16:8565. doi: 10.1038/s41598-026-43117-3 (PMC12976106; doi:10.1038/s41598-026-43117-3)
Supplement: Supplementary file 1 — Supplementary Material 1 [file 41598_2026_43117_MOESM1_ESM.pdf]

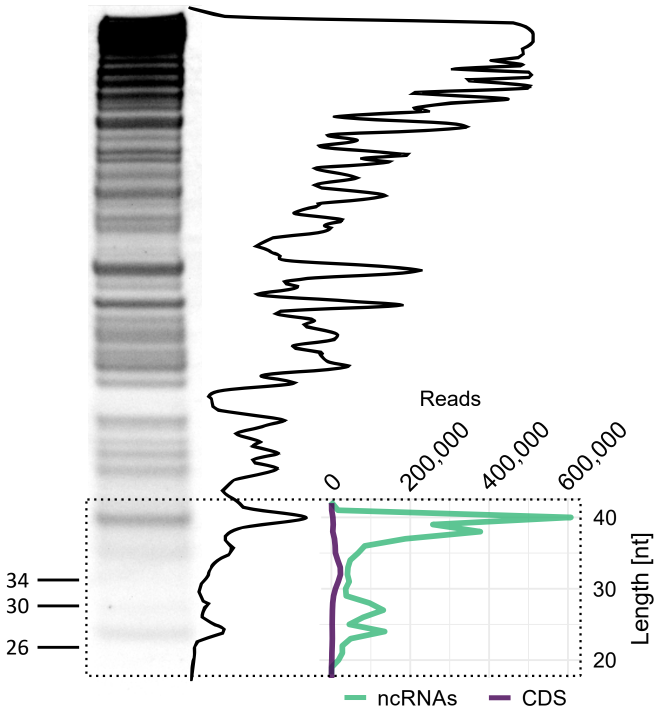

Supplementary Figure S1: RNA species breakdown of RPF bands during footprint generation quality control. Polysomes from 5-dag old Arabidopsis seedlings were digested with 0.3 U/µg$_{RNA}$ RNase If for 30 minutes at 21 °C and separated by two-phase urea-PAGE(12 + 18 % polyacrylamide, 7 M urea). Ribosome protected fragments were excised from the dotted region, sequenced, and analyzed with RiboSeQC. The graph to the right of the lane is the signal intensity of the bands. The fragment bands in the dotted region, which are regarded as quality control indicators for RPF generation, exclusively consist of non-coding RNAs (ncRNAs). The actual ribosomal footprints from coding sequences on translated mRNA (CDS) can not be differentiated from background noise or overlapping ncRNA signal in the gel picture.

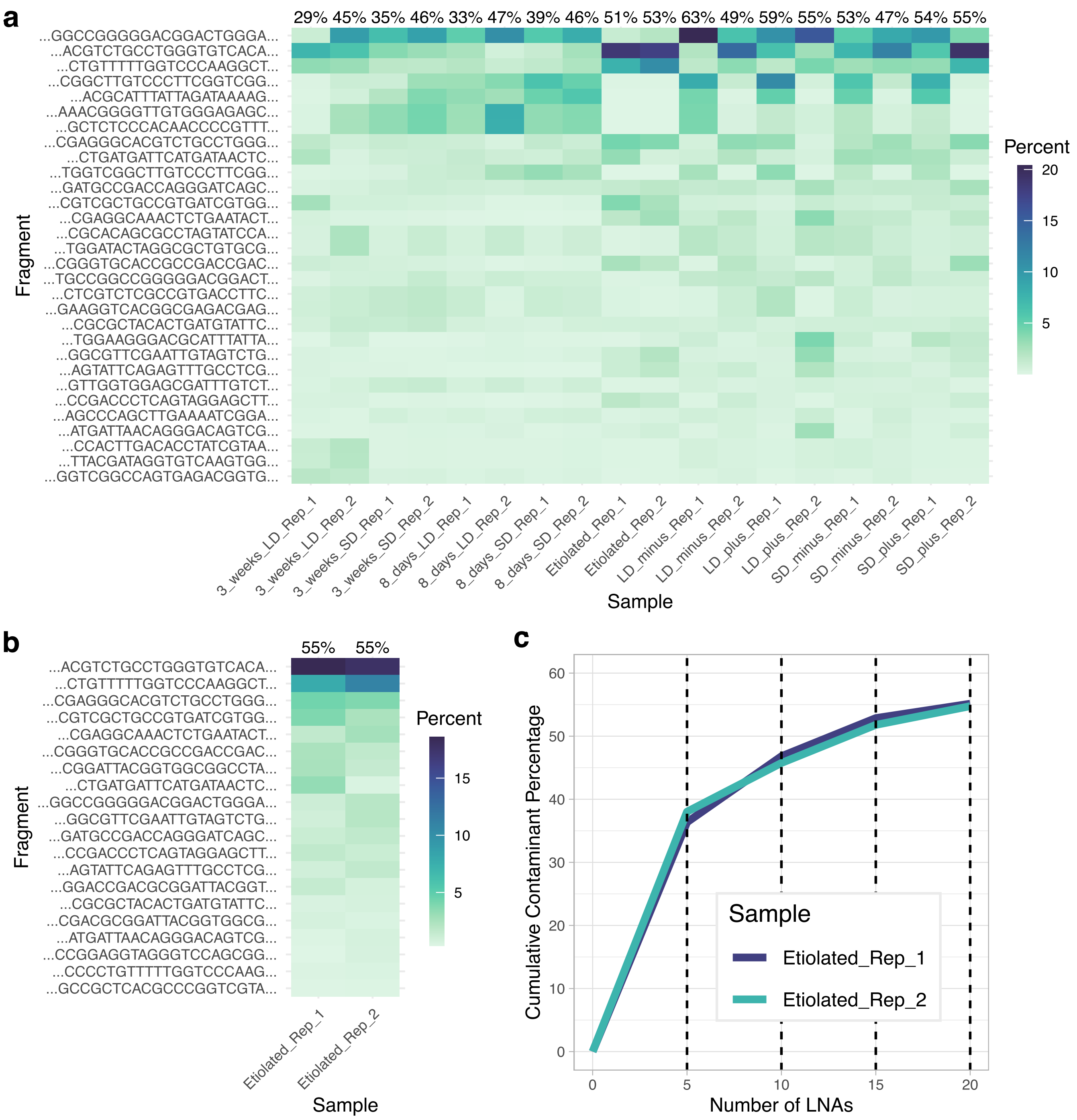

Supplementary Figure S2: Visualization of contaminant profiles across different growth conditions. Arabidopsis seedlings were grown for 8 days or 3 weeks in soil or hydroponically for 8 days in ½ MS medium with and without sucrose (plus/minus). Light conditions were either short-day (SD) or long-day (LD). One set of plants was grown hydroponically for 8 days in ½ MS medium without sucrose in total darkness (etiolated). RNase If was used for the footprinting reaction. a: Heatmap visualization of the 30 most abundant sequences after analysis with our script. Sorted by average abundance across all conditions. Each row represents a group of similar sequences of varying lengths, sharing a shortest common sequence, which is displayed as the y-axis label. Tile color represents the percentage of sequences in that group in relation to the total number of sequences in the sample. The cumulative percentage of the grouped sequences for each sample is indicated at the top of each column. b: Heatmap visualization of the 20 most abundant sequences in a single condition (etiolated seedlings). c: Figure of merit for estimating the optimal number of LNA targets for etiolated seedlings. Percentages of each target are added by walking down the sequences from top to bottom. Results are binned every 5 targets.

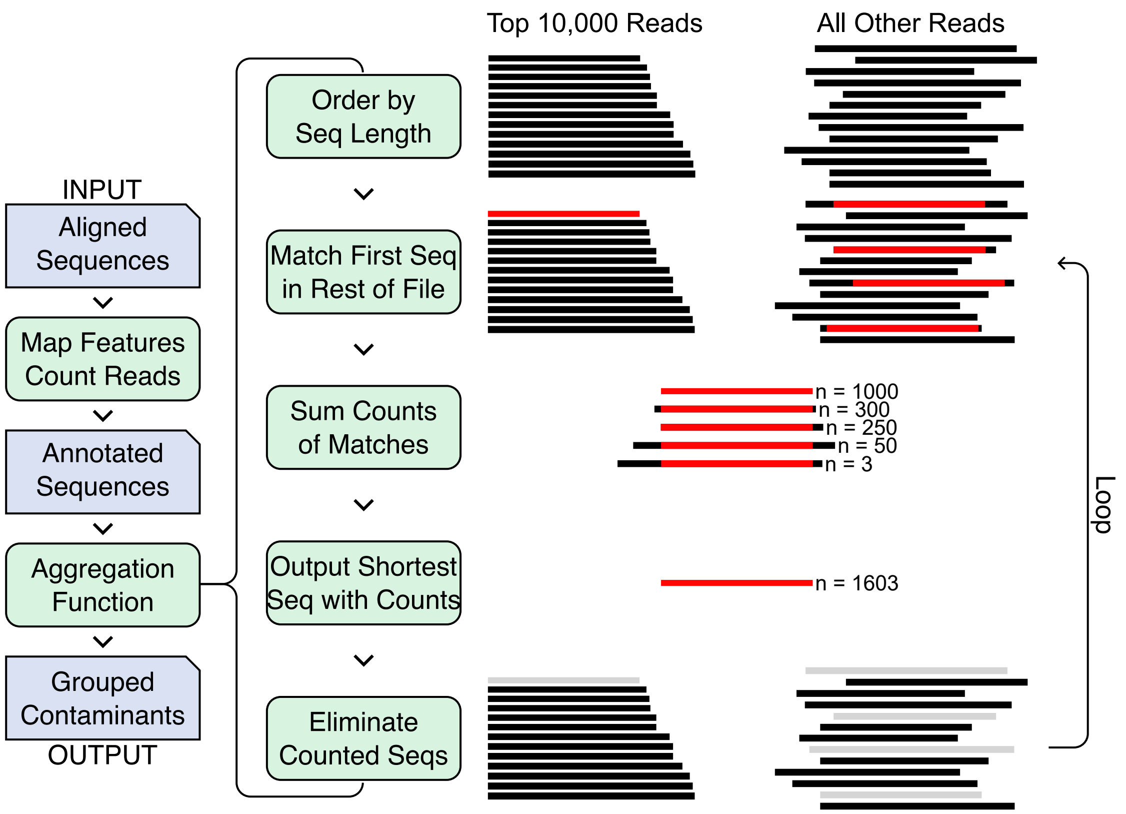

Supplementary Figure S3: Flow of the contaminant identification script. Alignments in BAM format are used as input for our contamination identification script in R (version 4.0 or later). First, aligned reads are mapped to the features from the supplied annotation file and identical reads are collapsed into single reads with a count using the packages Rsamtools, GenomicFeatures, and txdbmaker. The reads are then sorted by their abundance and the first 10,000 are selected as matching patterns. These reads are sorted by length and the shortest is matched throughout the entirety of all remaining reads via a "grep" command. All matching reads are grouped, and their summed counts as well as the shortest common sequence in the set are extracted as a result. All matched reads are eliminated from the search set before the next shortest read is queued for matching. This increases computation speed. The final data frame, containing the shortest sequence patterns and associated counts, is visualized using the ggplot2 package.

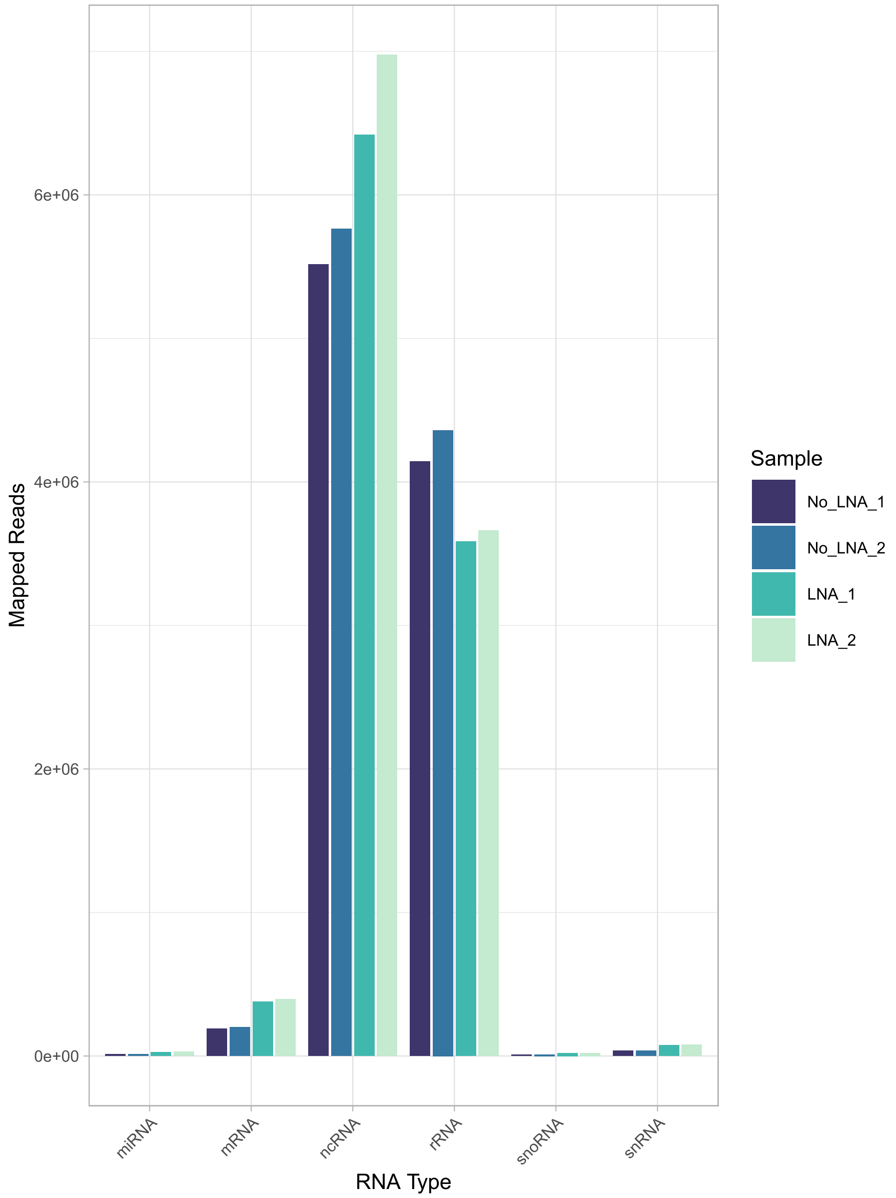

Supplementary Figure S4: Mapping of reads to various RNA species. Read mapping distribution reported by the quality control function of the ORFik R package for undepleted and LNA-depleted samples.
